# Suppressor mutations in *Escherichia coli* RNA polymerase alter transcription initiation but do not affect translesion RNA synthesis *in vitro*

**DOI:** 10.1016/j.jbc.2022.102099

**Published:** 2022-06-03

**Authors:** Nataliya Miropolskaya, Ivan Petushkov, Daria Esyunina, Andrey Kulbachinskiy

**Affiliations:** Institute of Molecular Genetics, National Research Center “Kurchatov Institute”, Moscow, Russia

**Keywords:** RNA polymerase, stringent response, translesion transcription, transcription-coupled repair, DksA, ppGpp, AP, apurinic/apyrimidinic site, εA, 1,N^6^-ethenoadenine, O6-meG, O6-methylguanine, RNAP, RNA polymerase, TCR, transcription-coupled repair, TEC, transcription elongation complex

## Abstract

Bacterial RNA polymerase (RNAP) coordinates transcription with DNA repair and replication. Many RNAP mutations have pleiotropic phenotypes with profound effects on transcription-coupled processes. One class of RNAP mutations (*rpo∗*) has been shown to suppress mutations in regulatory factors responsible for changes in gene expression during stationary phase or starvation, as well as in factors involved in the restoration of replication forks after DNA damage. These mutations were suggested to affect the ability of RNAP to transcribe damaged DNA and to decrease the stability of transcription complexes, thus facilitating their dislodging during DNA replication and repair, although this was not explicitly demonstrated. Here, we obtained nine mutations of this class located around the DNA/RNA binding cleft of *Escherichia coli* RNAP and analyzed their transcription properties *in vitro*. We found that these mutations decreased promoter complex stability to varying degrees, and all decreased the activity of rRNA promoters. However, they did not have strong effects on elongation complex stability. Some mutations were shown to stimulate transcriptional pauses or decrease intrinsic RNA cleavage by RNAP, but none altered the ability of RNAP to transcribe DNA templates containing damaged nucleotides. Thus, we conclude that the suppressor phenotypes of the mutations are unlikely to result from direct effects on DNA lesion recognition by RNAP but may be primarily explained by changes in transcription initiation. Further analysis of the effects of these mutations on the genomic distribution of RNAP and its interactions with regulatory factors will be essential for understanding their diverse phenotypes *in vivo.*

Transcription—the process of RNA synthesis on the genomic DNA template by RNA polymerase (RNAP)—is a pivotal step in gene expression. However, transcribing RNAP poses a major obstacle for other cellular machineries acting on the genomic DNA, first of all, during DNA replication (1, 2, 3). Replication–transcription conflicts can lead to replication stalling, replication fork collapse, and DNA damage in the case of both codirectional and head-on collisions of RNAP and the replisome, but the latter are apparently more deleterious to the cell (4, 5, 6, 7, 8, 9, 10). Most highly transcribed genes in bacteria, including rRNA operons, are co-oriented with replication, and their inversion leads to chromosomal damage and delays cell division (11, 12, 13, 14). One of proposed consequences of head-on collisions is replication fork reversal, during which the newly synthesized DNA strands anneal behind stalled forks. Reversed forks can be processed by the action of the Holliday junction resolvase RuvABC and the helicase-nuclease RecBCD, which likely remove the reversed double-stranded DNA end and restore the active fork geometry (1, 15).

Stalled transcription elongation complexes (TECs) represent a bigger challenge for the replisome in comparison with active TECs or promoter complexes (1, 7, 16, 17, 18). Transcriptional stalling can result from RNAP backtracking, which can by itself be provoked by conflicts with replication or impaired translation of nascent mRNA (17, 19, 20, 21, 22). Mutations of cellular factors involved in reactivation of stalled transcription complexes or their removal from the DNA template can greatly increase replication–transcription conflicts and associated DNA damage. These factors include Gre proteins that reactivate backtracked TECs by stimulating RNA cleavage in the active site of RNAP, accessory replicative helicases Rep and UvrD, the Mfd translocase that can disassemble stalled TECs, and the Rho factor of transcription termination (1, 4, 5, 6, 7, 17, 23, 24). R-loops formed during transcription, especially during head-on conflicts with replication, contribute to DNA damage, and their removal by RNaseH or helicase activities is essential for genome stability (1, 4, 10, 25). Various types of DNA lesions can also lead to RNAP stalling both *in vitro* and *in vivo*, and transcription complexes stalled on damaged DNA are a major threat to DNA replication. At the same time, transcribing RNAP acts as a sensor of DNA damage in the template strand, and stalled TECs recruit repair factors to DNA lesions during transcription-coupled DNA repair (TCR) (26, 27, 28).

Screening of suppressors of the UV-sensitive phenotype of *Escherichia coli* strains with defects in DNA repair and stringent response revealed a class of *rpo∗* mutations in the β and β′ subunits of RNAP that restored the viability of these strains under DNA damaging conditions and could also suppress mutations in the RuvABC resolvase (29, 30). Most of these mutations also suppressed defects in RecBCD involved in double-strand break processing, although with a lower efficiency (29, 30, 31). It was therefore proposed that the suppressor RNAP mutations may decrease conflicts with replication and prevent replication fork collapse and formation of Holliday junction intermediates, which require processing by RuvABC and RecBCD (15). Notably, *rpo∗* mutations could also suppress defects in the stringent response system and allow cell survival under stress conditions in the absence of the stringent alarmone ppGpp (29, 32). In WT cells, ppGpp and its cofactor DksA are responsible for changes in gene expression during starvation, by decreasing the synthesis of ribosomal RNA and proteins and stimulating transcription of biosynthetic operons (33). Limited analysis of *in vitro* properties of selected *rpo∗* RNAP variants and additional stringent RNAP mutants demonstrated that they decrease the stability of promoter complexes, including rRNA promoters (30, 31, 34, 35). Destabilization of promoter complexes by the suppressor mutations was proposed to explain their stringent phenotype, by mimicking the effects of ppGpp/DksA.

It was also suggested that *rpo∗* mutations may possibly change RNAP properties during transcription elongation (30, 31). In particular, it was hypothesized that the *rpo∗* mutations may destabilize the TEC and/or help the replication and repair factors to disassemble transcription complexes in highly transcribed or damaged DNA loci (30, 31). In support of this, some *rpo∗* mutations were shown to suppress deletions of the Rep and UvrD helicases that play an accessory role in replication by removing roadblocks to the replisome (23, 24), as well as deletions of the Mfd translocase and Gre factors (30). However, no detailed analysis of the transcriptional properties of various suppressor mutations has been performed to date, and their actual effects on the activity of RNAP have largely remained unknown.

To get better insight into the nature of the phenotypes of the suppressor *rpo∗* mutations, we obtained nine of these mutations in *E. coli* RNAP: Q148P, H447P, T563P, H1244Q, and G1260D substitutions in the β subunit and K215 E, Δ312-314, K789Q, and R1148H variants in the β′ subunit of *E. coli* RNAP (Fig. 1). All selected substitutions face either the downstream DNA binding channel (H447P, K215E, Δ312–314, and R1148H) or the RNA-DNA hybrid (Q148P, T563P, H1244Q, and G1260D); K789Q is located in the bridge helix contacting the template DNA strand and the RNA 3′-end at the active site (30).We purified the mutant enzyme variants, tested their properties *in vitro,* and showed that some of them moderately affect RNA elongation, by changing the efficiency of transcriptional pausing or intrinsic RNA cleavage, but none has significant effects on transcription of damaged DNA *in vitro*. At the same time, all the mutants decrease the activity of rRNA promoters *in vitro* and decrease the stability of promoter complexes to varying degrees. The results suggest that the suppressor effects of the RNAP mutations are unlikely to result from their direct influence on DNA lesion recognition or bypass but might be explained by changes in RNAP interactions with regulatory factors during RNA elongation or by their effects on transcription initiation and genomic distribution of RNAP.

**Figure 1:**
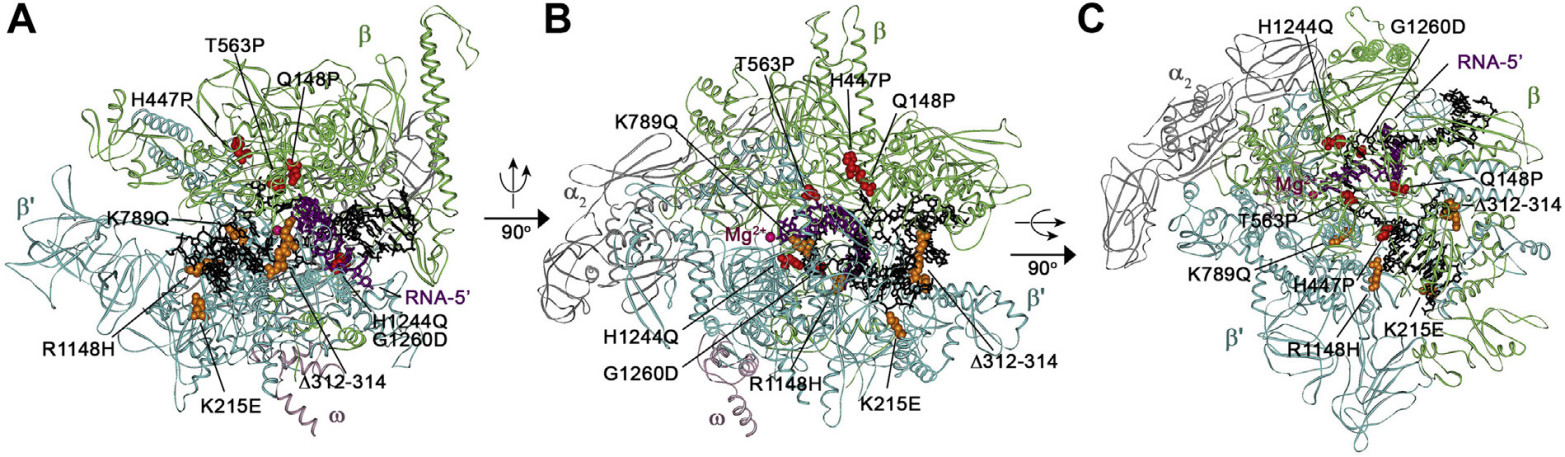
**Location of the analyzed suppressor mutations on the three-dimensional structure of the TEC of *E. coli* RNAP.***A*, view from the main cleft of RNAP (PDB: 6ALH (80)). *B*, view from the secondary channel. *C*, view from the top of the β subunit. Part of the melted segment of the nontemplate strand is disordered. The β, β′, α, and ω subunits are *light green*, *turquoise*, *gray,* and *light pink*, respectively; DNA is *black*, RNA is *violet*, catalytic Mg^2+^ is *pink*. Amino acid residues affected by the suppressor mutations in the β and β′ subunits are *red* and *orange*, respectively. RNAP, RNA polymerase; TEC, transcription elongation complex.

## Results

### Varying effects of RNAP mutations on transcription initiation

**Figure 2:**
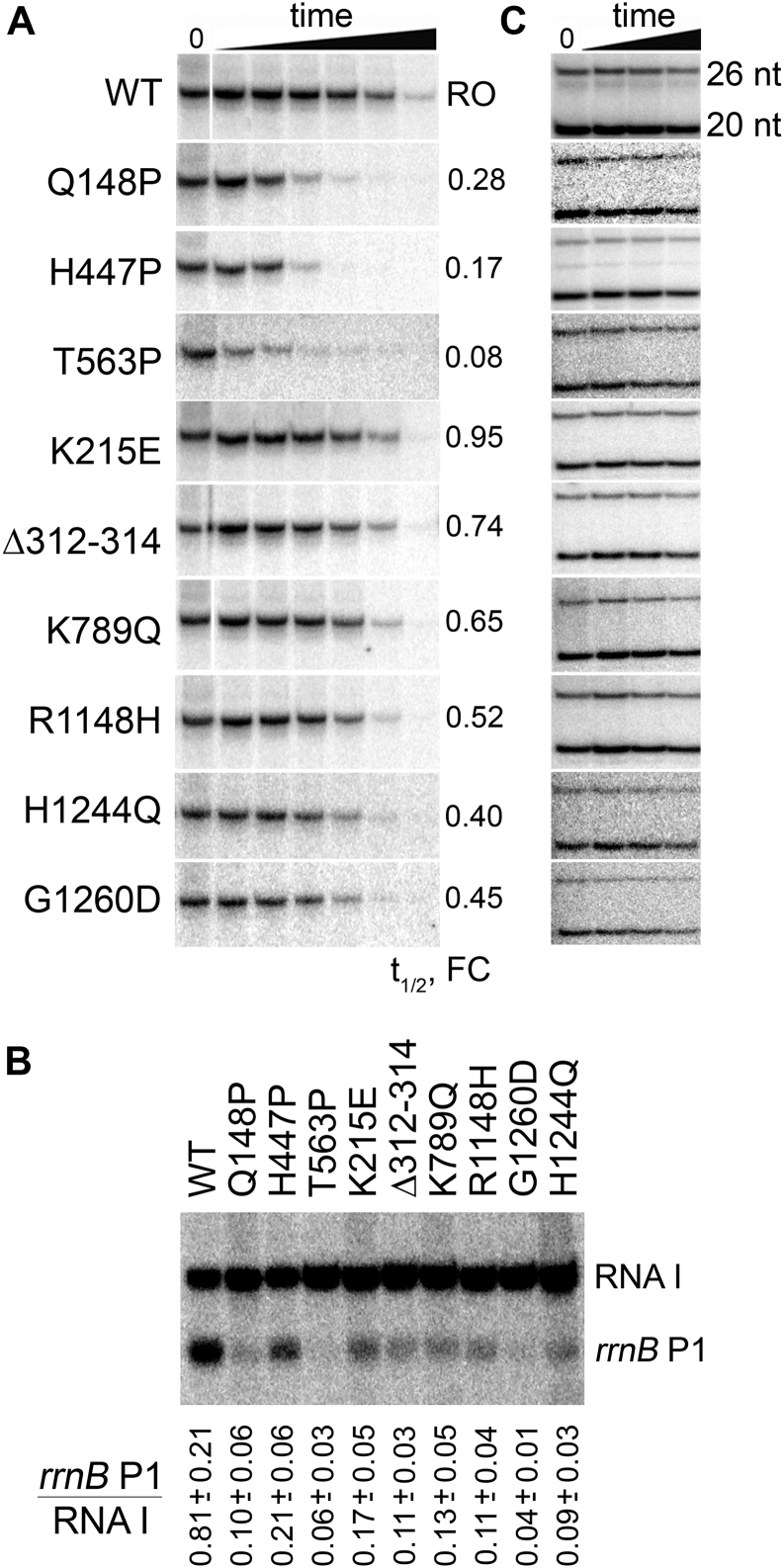
**Stability of transcription complexes formed by the mutant RNAPs.***A,* dissociation kinetics of T7A1 promoter complexes of WT and mutant RNAPs in the presence of heparin. RNAP activity was measured at increasing time intervals (0.5, 1, 2, 4, 10, and 30 min) after heparin addition by analyzing full-length (run-off, RO) RNA synthesis. Changes in the half-life times of promoter complexes of mutant RNAPs relative to WT RNAP are shown on the right (FC, fold-change). One of two independent experiments is shown (see Fig. S3 for the second replica). In the WT and K789Q panels, the gels are spliced to remove an extra lane. *B*, activities of WT and mutant RNAPs on supercoiled plasmid DNA containing the *rrnB* P1 and RNA I promoters. The ratio of full-length RNA products synthesized from the *rrnB* P1 and RNA I promoters is shown below the gel (means and standard deviations from three independent experiments). *C*, stability of the TECs formed by mutant RNAPs. The experiment was performed one or two times for different mutants. The complexes containing radiolabeled 20-mer and 26-mer stalled RNA transcripts were bound to an affinity resin, and the bound fraction was analyzed after incubation of the samples in the presence of 1 M KCl (for 0, 3, 10, 30 min). RNAP, RNA polymerase; TEC, transcription elongation complex.

Previously, several suppressor mutations in RNAP, including T563P analyzed here, were shown to decrease stability of promoter complexes, possibly explaining their stringent phenotype (34, 35). It was proposed that all *rpo∗* mutations should likely destabilize promoter complexes (30, 31), but only a few of them were directly tested *in vitro*. We therefore compared the effects of the nine RNAP mutations on promoter complex stability *in vitro*. We challenged promoter complexes formed by the WT or mutant RNAPs on the T7A1 promoter with heparin and measured RNAP activities after increasing time intervals (see Materials and Methods and Fig. S1 for all experimental details). Under the conditions of our experiments, about half of promoter complexes of WT RNAP dissociated within 5 min (the half-life time t_1/2_ = 4.2 ± 1.8 min; Figs. 2*A* and S3). The previously studied T563P substitution, as well as Q148P and H447P substitutions in the β subunit, greatly destabilized promoter complexes, with most complexes inactivated within 1 min or less (t_1/2_ was decreased 4–10-fold in comparison with WT RNAP) (Fig. 2*A*). The R1148H, H1244Q, and G1260D substitutions in the β subunit decreased t_1/2_ of promoter complexes 2- to 2.5-fold, while the remaining three RNAPs, K215E, Δ312-314, and K789Q in the β′ subunit had only minor effects on promoter complex stability (Figs. 2*A* and S3).

We further tested the activity of the mutant RNAPs on rRNA promoters, which form highly unstable promoter complexes (36), using a supercoiled plasmid containing the *rrnB* P1 promoter. The plasmid also contained a second promoter responsible for synthesis of RNA I from the replication origin, which forms stable complexes with RNAP (37). It was found that the activity of the *rrnB* P1 promoter relative to the RNA I promoter was significantly decreased for all mutant RNAPs in comparison with the WT enzyme (4–18-fold, *p*-value < 0.05 in all cases) (Fig. 2*B*).

It can therefore be concluded that various suppressor mutations decrease the stability of promoter complexes to varying degrees, but all have a common effect on the activity of rRNA promoters and possibly of other promoters that form unstable complexes with RNAP.

### No effects of RNAP mutations on RNA elongation and intrinsic termination

Previous studies of suppressor mutations in *E. coli* RNAP proposed that they might destabilize TECs formed by the mutant RNAPs thus helping the replisome and repair factors to displace them from the DNA template (see Introduction). To reveal possible effects of these mutations on transcription elongation, we compared the TEC stabilities, the rates of RNA synthesis, and the efficiency of intrinsic transcription termination by the WT and mutant RNAP variants.

We first tested the stabilities of TECs formed by the mutant RNAPs obtained after transcription initiation and stalled at the +20 template position by nucleotide deprivation. The TECs were bound to affinity resin and incubated at high ionic strength conditions to induce RNA dissociation, and the fraction of stably bound–labeled RNA transcripts was measured over time (Fig. 2*C*). It was found that the majority of the complexes formed by WT *E. coli* RNAP remained bound to the sorbent during the course of experiment. Similarly, the analyzed RNAP substitutions did not result in dramatic changes in the TEC stability, with the major fraction of RNA remaining bound to the TEC within 30 min. Therefore, the mutations do not have great destabilizing effects on the TEC, at least in the absence of additional factors.

**Figure 3:**
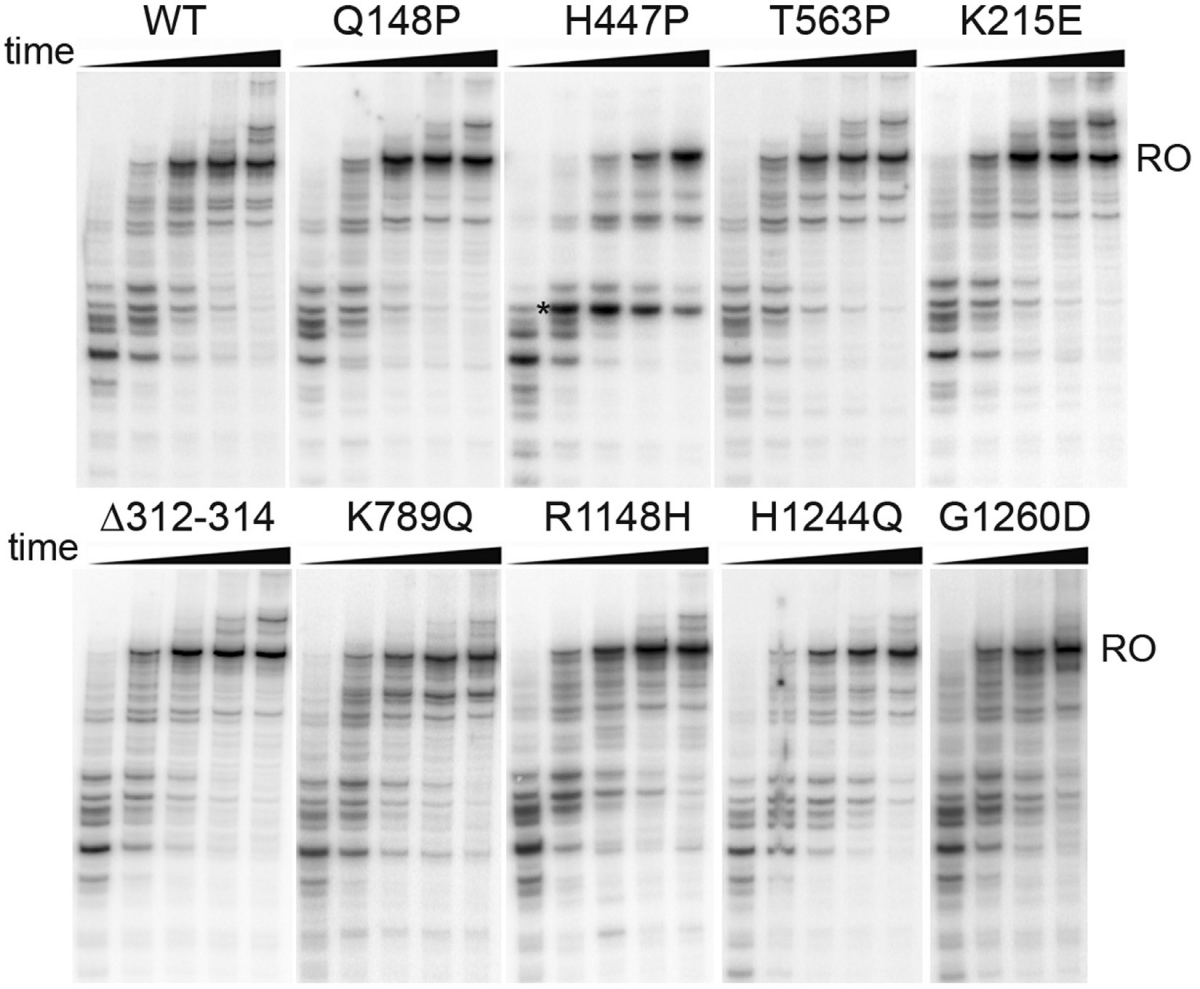
**Transcription elongation by the mutant RNAPs.** The kinetics of full-length RNA synthesis (run-off, RO) was measured on a DNA template containing the λP_R_ promoter fused to a fragment of the *rpoB* gene lacking strong pausing signals, after the addition of NTP substrates to stalled 26-mer complexes at 20 °C (for 0.5, 1, 2, 3, 5 min for most RNAPs; for 0.5, 1, 2, 3 min for G1260D RNAP). Transcriptional pause stimulated by the H447P substitution is indicated with an *asterisk*. The experiment was performed one time for each mutant; a similar experiment performed in the presence of DksA and ppGpp is shown in Fig. S4. RNAP, RNA polymerase.

To determine the average rate of RNA elongation, we analyzed the kinetics of RNA extension in transcription complexes formed on a DNA template containing a 500 bp long fragment of the *rpoB* gene lacking strong pause-inducing signals (Fig. 3) (38). For the WT RNAP, the synthesis of the full-length run-off RNA product was detected starting from the first minute of the reaction. A similar kinetics of RNA synthesis and a similar pattern of shorter RNA bands likely corresponding to transient transcriptional pauses were observed for the mutant RNAP variants (Fig. 3). Furthermore, the addition of DksA and ppGpp did not have major effects on the kinetics of RNA synthesis on this template for both WT and mutant RNAP variants (Fig. S4). An exception was the H447P RNAP that showed a delayed kinetics of full-length RNA synthesis both in the absence and in the presence of DksA/ppGpp, apparently by stimulating some transcriptional pauses (Figs. 3 and S4). Therefore, most suppressor mutations do not strongly affect the rate of undisturbed RNA elongation by bacterial RNAP.

**Figure 4:**
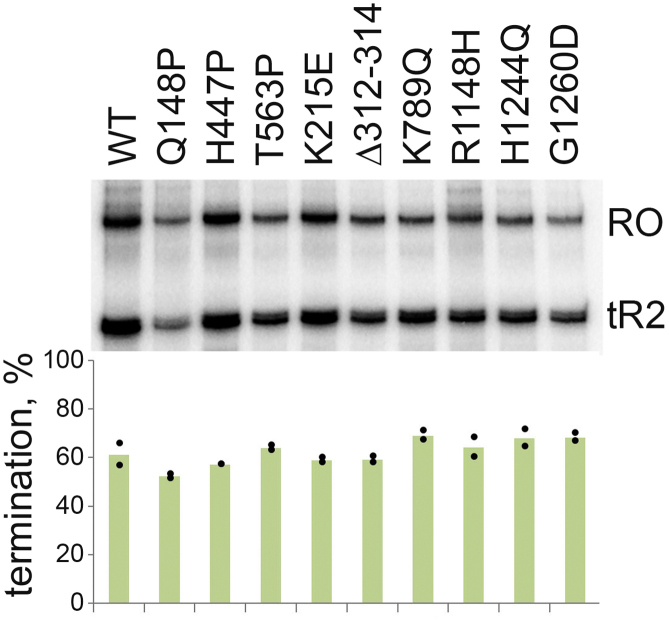
**Transcription termination by the mutant RNAP.** The efficiency of transcription termination was measured on the λ tR2 terminator and defined as the ratio of the terminated and run-off (RO) RNA products. Means from two independent measurements are shown. RNAP, RNA polymerase.

If the suppressor mutations destabilized the TEC, it could be expected that they would increase the efficiency of intrinsic transcription termination, by stimulating RNAP dissociation. To determine whether this was the case, we compared the efficiency of intrinsic transcription termination by the WT and mutant RNAPs on the λ tR2 terminator (Fig. 4). It was found that all RNAP variants had comparable levels of termination. Therefore, the analyzed mutations are unlikely to have a general destabilizing effect on transcription complexes.

### Moderate effects of some RNAP mutations on transcriptional pausing

**Figure 5:**
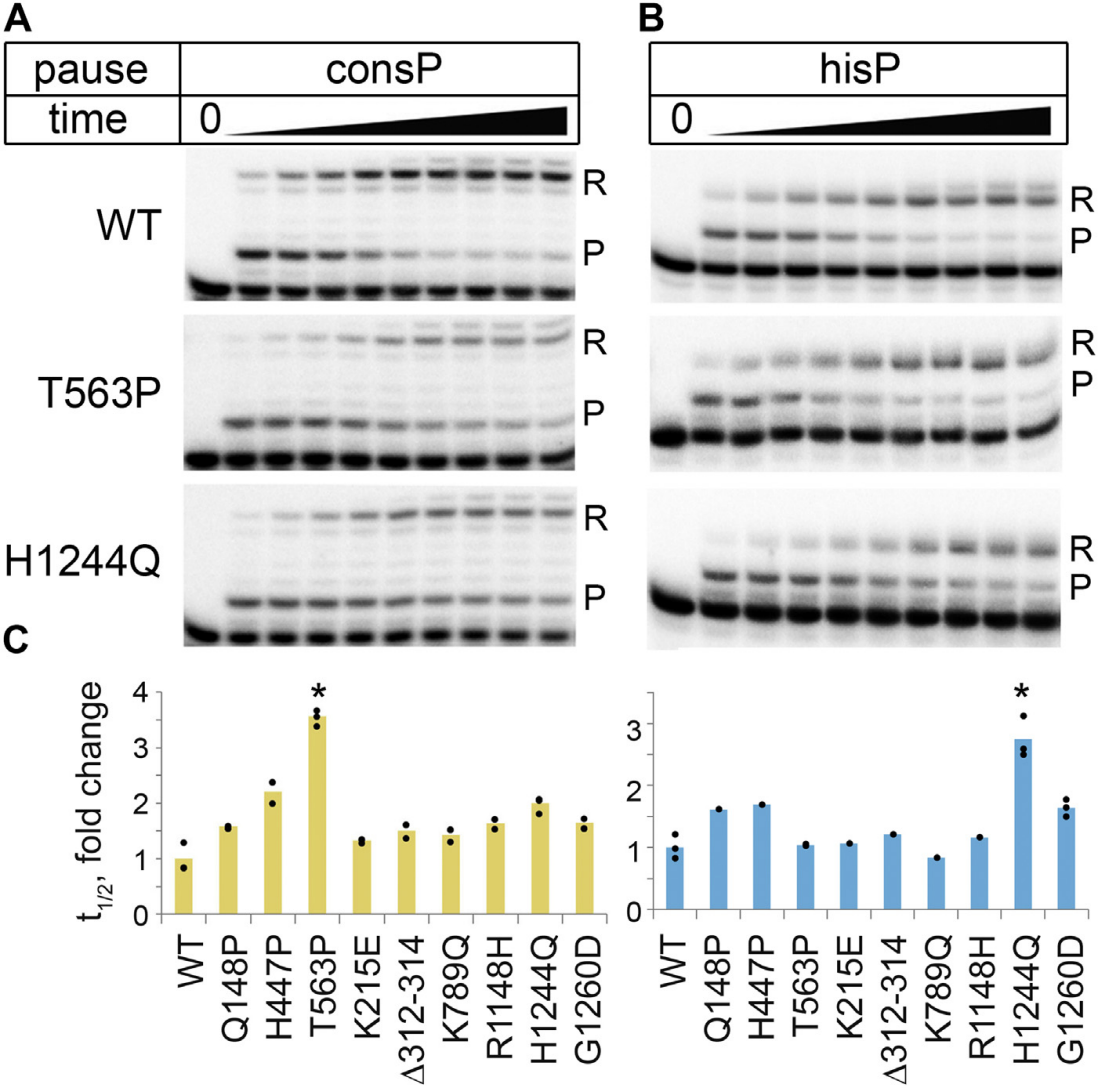
**Effects of RNAP mutations on transcriptional pausing.***A*, the kinetics of pausing at the consP pause signal for WT, T563P, and H1244Q RNAPs (5”, 15”, 30”, 1′, 2′, 5′, 10′, 15′, 30′ after NTP addition to pre-assembled TECs). *B*, pausing at the hisP pause signal (5”, 15”, 30”, 1′, 2′, 4′, 10′, 15′, 30′ after NTP addition). The sequences of reconstituted TECs used for analysis of pausing are shown in Fig. S2. Positions of the paused (P) and read-through (R) RNA products are indicated. *C*, changes in the pause half-life times for the mutant RNAPs relative to the WT control (the results from 1-3 independent measurements; see Table S1 for individual data points). Statistically significant differences between the T563P and H1244Q mutants and WT RNAP are indicated (∗*p* < 0.05). RNAP, RNA polymerase; TEC, transcription elongation complex.

The observed effects of one of the analyzed substitutions on transcriptional pausing (H447P, see above) prompted us to investigate the effects of the suppressor mutations on the recognition of site-specific pausing signals by RNAP. We first tested whether any of the mutations can change the duration of the elemental transcriptional pause revealed in genome-wide studies of RNAP pausing (39, 40, 41). These studies identified the consensus pause sequence containing the conserved G_-10_(C/T)_-1_G_+1_ signal, surrounded by additional less conserved motifs (39). We assembled TECs on the consensus pause (consP) template and monitored the kinetics of pausing after the addition of nucleotides (Figs. S1 and S2). The half-life time of the pause for WT RNAP measured under our conditions was 22 ± 6 s (Fig. 5*A* and Table S1). Most mutant RNAPs paused with comparable kinetics (≤1.5-fold changes in the pause t_1/2_). At the same time, for three RNAPs, the pause duration was increased about 2-fold (H447P and H1244Q) or 3.5-fold (T563P) relative to the WT enzyme (*p* < 0.05) (Fig. 5*A* and Table S1).

Next, we analyzed the kinetics of RNAP pausing on another well-studied pause signal, hairpin-dependent pause from the histidine operon attenuator (hisP), by assembling the TEC on a synthetic template containing the hisP sequence. Formation of the upstream RNA hairpin required for stabilization of the pause was mimicked by the addition of a short RNA oligonucleotide complementary to the RNA transcript in the complex (Figs. S1 and S2) (42, 43). The hisP pause half-life time for WT RNAP was 44 ± 9 s (Fig. 5*B*). Most analyzed RNAP mutations, including T563P that stimulated consP pausing, did not strongly affect hisP pausing (≤1.5-fold changes in the pause t_1/2_) (Fig. 5*B* and Table S1). However, the H1244Q substitution increased the hisP pause duration about 3-fold (t_1/2_ = 120 ± 15 s). A similar stimulatory effect of this mutation on hisP pausing was observed on a DNA template containing the native hisP signal during promoter-dependent transcription (Fig. S5). Therefore, most suppressor mutations in RNAP do not have strong effects on site-specific pausing, in agreement with the absence of their effects on the average rate of RNA elongation. At the same time, some of these substitutions can stimulate elemental or hairpin-dependent pausing, with T563P and H1244Q having the strongest effects on these two types of pauses.

### Minor effects of RNAP mutations on RNA cleavage and mismatch extension

The process of RNAP backtracking during the elongation step of transcription plays important roles in the regulation of gene expression and in the maintenance of genome stability. Backtracked complexes are reactivated by RNA cleavage in the active site of RNAP, which can be stimulated by the secondary channel factors GreA and GreB (44, 45). RNAP backtracking can be stimulated by nucleotide misincorporation during RNA elongation, and RNA cleavage in such complexes is the main mechanism of transcriptional proofreading (41, 46, 47). Stalled backtracked complexes can provoke conflicts of transcription with DNA replication resulting in DNA damage but at the same time can stimulate repair of double-strand breaks in DNA (7, 17, 48, 49). Since the suppressor mutations increase cell survival under DNA damaging conditions, we tested whether they can affect nucleotide misincorporation, TEC backtracking, and RNA cleavage.

We first analyzed possible effects of the mutations on the ability of RNAP to misincorporate a noncomplementary nucleotide in a TEC containing fully complementary RNA transcript (3′-rA opposite template dT, rA-dT) (Figs. S1 and S2). The WT RNAP and all the mutants had almost identical patterns of RNA extension in this reaction (Fig. S6*A*). Furthermore, we tested the effects of the mutations on RNA extension in a mismatched TEC containing a noncomplementary nucleotide at the RNA 3′-end (3′-rA opposite template dG, rA-dG). The mutant RNAPs also did not differ from the WT enzyme in the pattern of RNA extension (Fig. S6*B*). Therefore, the mutations do not change the fidelity of RNAP in these reactions.

**Figure 6:**
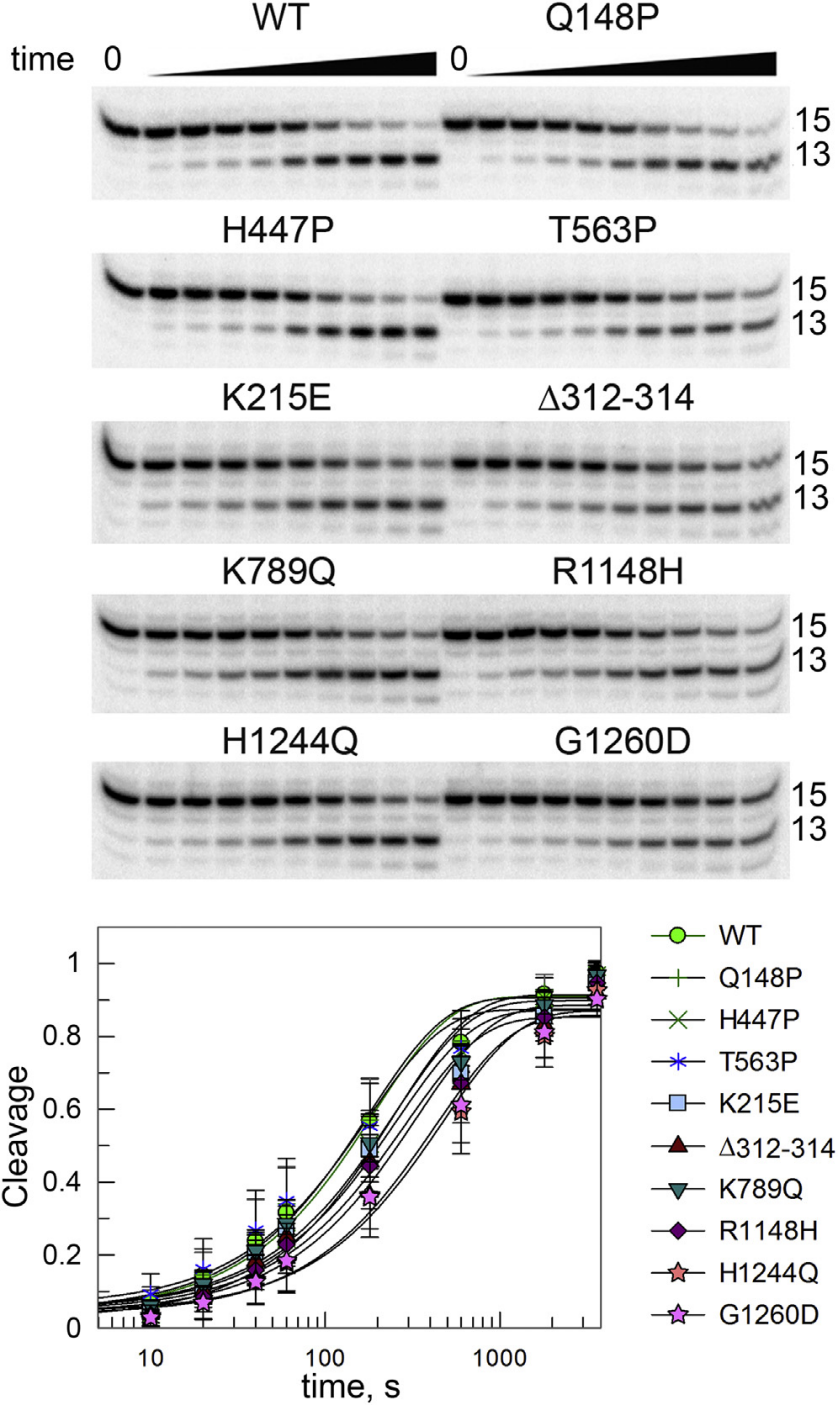
**Intrinsic RNA cleavage by the WT and mutant RNAPs in a mismatched TEC.** The rA-dG TECs containing RNA transcript with an unpaired 3′-RNA adenine nucleotide were reconstituted from synthetic oligonucleotides (Fig. S2). The starting 15-mer RNA and the 13 nt cleavage product are indicated. The plot shows the kinetics of the cleavage reaction for each RNAP (means and standard deviations from three independent measurements; see Table S1 for *k*_obs_ values). RNAP, RNA polymerase; TEC, transcription elongation complex.

To measure the rate of intrinsic RNA cleavage by the mutant RNAPs, we assembled the mismatched rA-dG TEC and analyzed the kinetics of the reaction after the addition of Mg^2+^ ions in the absence of nucleotides (Fig. 6). Most of the mutations did not greatly affect RNA cleavage in comparison with WT RNAP, but substitutions H1244Q and G1260D decreased the cleavage rate ∼3 fold (*p*-value <0.05) (Fig. 6 and Table S1). Thus, suppressor mutations in general do not change the RNA cleavage activity of RNAP in mismatched complexes, although some of them may decrease the efficiency of RNA cleavage and/or RNAP backtracking. In agreement with this, the H1244Q substitution was shown to decrease formation of stalled backtracked complexes during transcription elongation (7).

### No effects of mutations on translesion RNA synthesis

**Figure 7:**
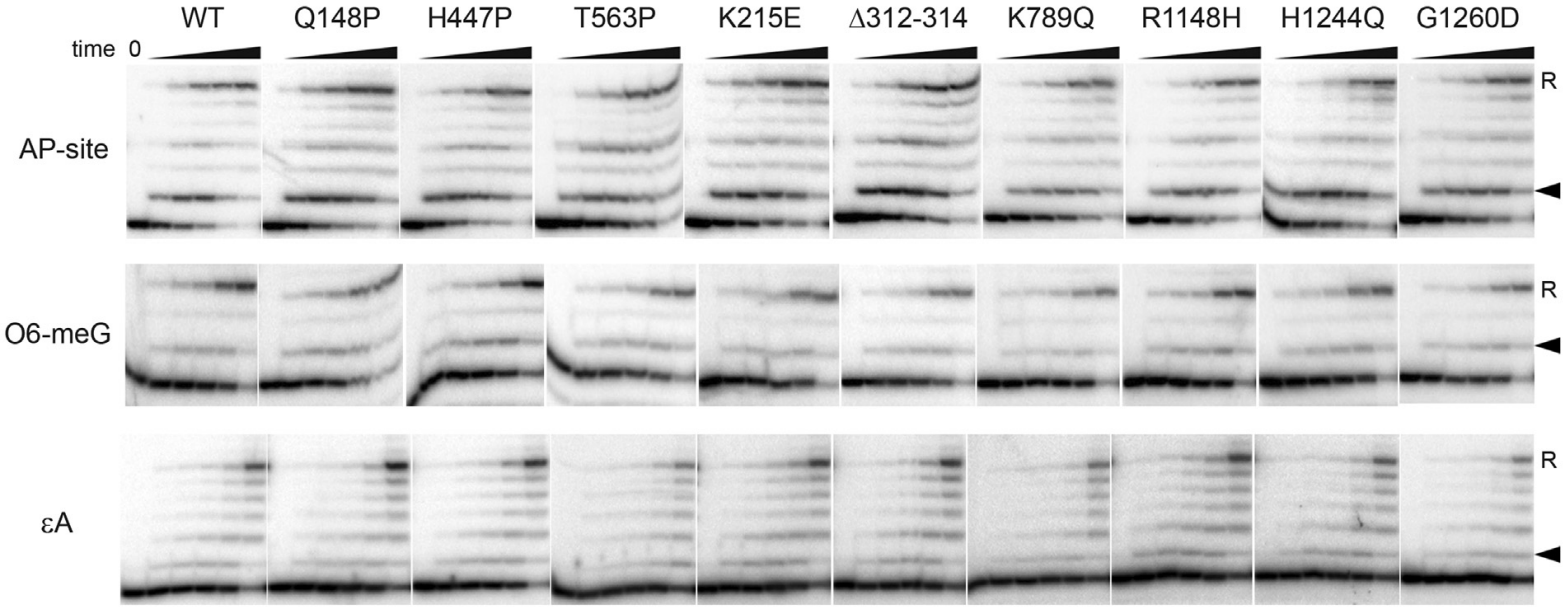
**Kinetics of RNA synthesis on damaged DNA templates by the WT and mutant RNAPs for AP (top), O6-meG (middle), and εA (bottom) templates**. The TECs containing damaged nucleotides and corresponding control TECs were reconstituted from synthetic oligonucleotides (Fig. S2). Positions of damaged template nucleotides are indicated with *arrowheads*. For each TEC, an incomplete set of NTPs was added, resulting in limited extension of the RNA transcript (read-through, R) that was stalled beyond the lesion. The reactions were performed for 10”, 30”, 1′, 3′, and 30’. Representative gels from two independent experiments are shown. RNAP, RNA polymerase; TEC, transcription elongation complex.

During initial characterization of the suppressor mutations in RNAP, it was proposed that they may change the ability of RNAP to transcribe damaged DNA and potentially affect the stability of transcription complexes stalled at DNA lesions (29, 30). Therefore, we analyzed transcription of DNA templates containing various types of damaged nucleotides by the mutant RNAPs. We reconstituted TECs using template DNA oligonucleotides containing the apurinic/apyrimidinic site (AP-site), O6-methylguanine (O6-meG), or 1,N6-ethenoadenine (εA) at a defined position one nucleotide downstream of the RNA 3′-end (Figs. S1 and S2). We measured the kinetics of RNA extension on damaged templates (Fig. 7) in comparison with corresponding control templates (Fig. S7). The reactions were performed in the presence of an incomplete set of NTPs to allow RNA extension to a position several nucleotides downstream of the lesion on each template (Figs. S1 and S2).

It was found that on the control undamaged templates, all RNAPs rapidly extended the RNA transcript to the expected position (the readthrough RNA product was synthesized within the first 10 s of the reaction) (Fig. S7). The kinetics of RNA extension was strongly delayed in the case of all three lesions. In agreement with published data (50, 51, 52, 53), on the AP-site template, RNAP was stalled at two positions, one nucleotide upstream and directly opposite of the lesion, and further RNA synthesis was significantly delayed (Fig. 7*A*). O6-meG had a similar effect on RNA extension, with a stronger pause observed upstream of the lesion and a weaker pause opposite the lesion (Fig. 7*B*) (50). εA strongly inhibited nucleotide incorporation opposite the lesion, with the major pause observed upstream of the lesion (Fig. 7*C*) (50, 51, 53). Remarkably, none of the mutations led to dramatic changes in the kinetics of RNA synthesis and the pattern of extended RNA products on both control and damaged templates.

Finally, we tested whether DksA and ppGpp may affect RNA synthesis on damaged DNA templates by the WT or mutant RNAPs. The addition of both factors only slightly inhibited RNA extension on the AP-site template by the WT RNAP (Fig. S8), in agreement with published data showing weak effects of DksA/ppGpp on translesion synthesis by WT *E. coli* RNAP (53). Similarly, DksA and ppGpp had only minor effect on RNA extension opposite the AP-site by all mutant RNAPs (Fig. S8). Overall, it can be concluded that none of the tested suppressor mutations has strong effects on the recognition of DNA lesions and on the efficiency of translesion RNA synthesis by *E. coli* RNAP *in vitro*.

## Discussion

Transcription-replication conflicts are a major source of chromosomal DNA damage in bacteria. Previous studies revealed the central role of RNAP in this process as well as in TCR (see Introduction). Genetic screenings identified *rpo∗* mutations in *E. coli* RNAP that could suppress the UV-sensitive phenotype of *ruv-*minus and *recB-*minus strains (29, 30); the best studied mutation of this class is *rpo*∗35, corresponding to the H1244Q substitution in the β subunit, included in our analysis. All *rpo∗* alleles also have the stringent phenotype and suppress the absence of the stringent response alarmone ppGpp (29, 30, 32). Several of the *rpo∗* mutations, including H1244Q, restore viability of strains lacking Gre factors, the Mfd translocase, or the accessory helicases Rep and UvrD (23, 24, 30, 31). Recently, Rep, UvrD, and Mfd were shown to directly remove stalled TECs that serve as barriers for the replisome *in vitro* (5)(6). The H1244Q and G1260D mutations also suppressed transcription-replication conflicts exacerbated upon deletion of the Rep helicase and expression of deinococcal helicase RecD2 (54) or upon overexpression of the replicative helicase DnaB (55). It was therefore hypothesized that the *rpo∗* mutations may decrease transcription-replication conflicts by destabilizing TECs formed by the mutant RNAPs, including transcription of damaged DNA (30, 31). Here, we have analyzed nine selected *rpo∗* mutations, located in the nucleic acid binding cleft of RNAP close to the downstream DNA duplex and the RNA/DNA hybrid (Fig. 1), in a series of *in vitro* transcription tests to reveal their possible effects on the transcription complex stability, transcriptional pausing and termination, fidelity of RNA synthesis, and transcription of damaged DNA templates.

All stringent RNAP mutations were proposed to decrease promoter complex stability but only a few of them were directly studied *in vitro* (30, 34, 35). We have found that several *rpo∗* mutations indeed decrease promoter complex stability on the moderately stable T7A1 promoter (Fig. 2*A*) (56). The strongest effects are observed for the Q148P, H447P, and T563 substitutions. Two of these substitutions, Q148P and T563P, face the rifampicin-binding pocket of the β subunit (Fig. 1) and confer resistance to rifampicin (30). Previously, the Q148P substitution was shown to decrease promoter complex formation on the λ *cro* promoter (30), and the T563P substitution decreased the activity of rRNA promoters (34). Furthermore, substitution H447P is located in the CRE pocket of the β subunit that interacts with the nontemplate DNA strand just downstream of the active site, mutations in which were shown to destabilize promoter complexes (57, 58). The destabilizing effects observed for the T7A1 promoter are nonuniform for various *rpo∗* mutants, suggesting that they may have varying effects on gene expression *in vivo*. At the same time, they all alleviate defects in the stringent response system, which is responsible for inhibition of rRNA promoters during starvation (29, 30, 34, 35). Intriguingly, the activities of most *rpo∗* mutants on rRNA promoters have never been tested. We have found that all nine analyzed mutants strongly decrease the activity of the *rrnB* P1 promoter (Fig. 2*B*). This is the single common property of the *rpo∗* mutants that we have studied, which may possibly explain their common phenotypes observed *in vivo* (see below).

Previously, the *rpo∗* mutations were proposed to decrease stability of TECs (30, 31). In particular, two suppressor mutations in RNAP, H551P, and H1244Q, were shown to decrease formation of stalled TEC arrays when transcription was stalled by nucleotide deprivation or by using damaged DNA templates (31). We have observed that the nine *rpo∗* mutations tested here, including H1244Q, do not change the rate of transcription elongation (Fig. 3) and do not increase the rate of TEC dissociation at high ionic strength conditions in comparison with WT RNAP (Fig. 2*C*). A similar result was obtained previously in another study of H1244Q RNAP (7). In comparison, mutations in the switch2 region connecting the clamp domain to the main RNAP body greatly increased the rate of TEC dissociation under the same conditions (59). Furthermore, none of the suppressor mutations change the efficiency of intrinsic transcription termination, suggesting that they do not destabilize the TEC in these assays (Fig. 4). It is therefore possible that the decrease in the formation of stalled TECs previously observed for some suppressor mutants (31) resulted from changes in transcription initiation but not in the elongation complex stability.

At the same time, some of the analyzed RNAP substitutions have specific effects on the TEC properties, likely reflecting their different positioning within the TEC. Several of the tested mutations, including H447P, T563P, and H1244Q in the β subunit, stimulate elemental or hairpin-dependent pausing (Fig. 5). The H447P substitution also slows down the kinetics of full-length RNA synthesis both in the absence and in the presence of DksA/ppGpp (Figs. 3 and S4). We propose that the H447P substitution located in the CRE pocket of core RNAP may potentially change its contacts with the nontemplate DNA strand downstream of the active site, similarly to an adjacent D446A substitution (40, 57, 58), thus affecting transcriptional pausing. Substitution T563P, located in fork-loop2 in the rifampicin-binding pocket, may change RNAP contacts with the first nucleotides of the RNA-DNA hybrid (Fig. 1). The H1244Q substitution (and adjacent G1260D) is located near the RNA-DNA hybrid in the TEC, not far from the RNA exit channel, and may therefore modulate hairpin-dependent pausing by affecting conformational changes in the TEC associated with the hairpin folding (60, 61).

The fidelity of transcription depends both on the fidelity of nucleotide misincorporation by RNAP and on its ability to extend mismatched RNA transcripts or perform RNA cleavage and proofreading (46, 62). Nucleotide misincorporation can interrupt the continuity of transcription and stimulate RNAP backtracking leading to deleterious consequences for genome stability (17, 41, 63, 64). The rate of RNA cleavage was shown to be different for RNAPs from different bacterial species, which may have potential regulatory roles in gene expression and DNA repair (65, 66, 67). Previously, mutations in the active site of RNAP and in regions surrounding the RNA-DNA hybrid were shown to increase transcriptional mutagenesis by decreasing nucleotide selectivity or stimulating transcript slippage (68, 69, 70, 71). In contrast, our analysis of the suppressor mutations revealed no changes in nucleotide misincorporation and mismatched RNA extension (Fig. S6). Furthermore, most analyzed mutations do not significantly change the rate of intrinsic RNA cleavage in mismatched TECs containing a noncomplementary nucleotide in the RNA 3′-end (Fig. 6). Two of the mutations, H1244Q and G1260D, decrease the RNA cleavage rate about 3-fold. This might be explained by stabilization of an inactive conformation of the TEC by the mutations (since the H1244Q substitution also increases the duration of site-specific transcriptional pauses) or their inhibitory effects on RNAP backtracking. Indeed, the H1244Q substitution was previously shown to decrease formation of stalled backtracked transcription complexes *in vitro* (7). However, in general, the suppressor phenotypes of mutations are unlikely to result from their effects on nucleotide misincorporation or on the intrinsic transcript cleavage activity of RNAP.

Previously, the *rpo∗* mutants were proposed to directly affect translesion transcription and destabilize TECs stalled on damaged DNA (30, 31). We have shown that none of the suppressor mutations affects translesion RNA synthesis by RNAP. It has been found that the kinetics of RNA extension and the patterns of transcriptional stalling are almost identical for the WT and mutant RNAPs variants on all three tested DNA lesions, 8-oxoG, O6-meG, and εA (Fig. 7). Furthermore, ppGpp and DksA have similarly weak inhibitory effects on RNA extension on damaged DNA templates by the WT and mutant RNAPs (Fig. S8). These observations argue against the possibility that the suppressor mutations may directly stimulate transcription opposite DNA lesions or prevent formation of stalled complexes on damaged DNA templates. Since previously published assays included analysis of both transcription initiation and elongation (31), the reported effects of the mutations on transcription of damaged DNA might have resulted from changes in transcription initiation by the mutant RNAPs.

Overall, these observations suggest that while having general effects on transcription initiation, the suppressor mutations do not dramatically compromise the intrinsic stability of the TEC during transcription elongation or termination and do not visibly affect translesion RNA synthesis. Some of them may specifically affect transcriptional pausing and RNA cleavage with potential outcomes for genetic regulation. These changes in the TEC properties may possibly result from specific effects of individual substitutions on RNAP interactions with DNA and RNA and/or conformational changes in the TEC but are unlikely to underlie the common suppressor effects of all *rpo∗* mutations.

Although the *rpo∗* substitutions are not a homogeneous group of mutations (30), several common mechanisms explaining their known phenotypes can be proposed. First, despite the absence of direct effects on translesion synthesis, these mutations might increase the sensitivity of the TEC to accessory factors that help to remove RNAPs stalled at DNA lesions or in backtracked complexes, such as the Rep and UvrD helicases acting during replication, Gre factors reactivating backtracked complexes, or the Rho factor and the Mfd translocase promoting transcription termination (6, 23, 24, 30, 50, 72, 73, 74). It was also proposed that the suppressor mutations may affect RNAP backtracking and formation of transcription-associated R-loops, as was shown for H1244Q (7, 75). However, whether this is also true for other suppressor mutations remains to be tested.

The *rpo∗* mutations might also affect transcription-repair coupling mediated by the Mfd translocase or the UvrD helicase (27, 28, 76, 77). In particular, the H1244Q mutation was shown to suppress the sensitivity of ppGpp-minus cells to genotoxic stress depending on the presence of UvrD (30, 31, 77) and was proposed to stimulate UvrD-dependent backtracking of the TEC during TCR (77). However, since the H1244Q substitution in various contexts can suppress deletions of both Mfd (30) or UvrD (23, 24) it is unlikely that this substitution and other suppressor mutations exert their effects solely in cooperation with these factors. Currently, it remains unknown whether other suppressor mutations may affect the RNAP function in transcription-repair coupling. Thus, further analysis is needed to discover possible effects of the RNAP mutations on the sensitivity of stalled TECs to the action of regulatory factors *in vitro* and *in vivo*.

Finally, we propose that the observed effects of the suppressor mutations on promoter complex stability and transcription initiation may by themselves be sufficient to explain their phenotypes *in vivo*, because of the reduced activity of rRNA promoters and global changes in gene expression. Changes in the rate of transcription initiation can greatly affect the density of elongating RNAPs and potentially decrease barriers to replication, thus explaining the suppressor effects of the *rpo∗* mutations on deletions of elongation factors and accessory helicases (23, 24, 30, 31). The major source of transcription-replication conflicts is the activity of rRNA operons, and ppGpp and DksA can decrease the deleterious consequences of such conflicts by suppressing the activity of rRNA promoters (32, 33). On the contrary, inversions of ribosomal operons greatly stimulate these conflicts, especially in rich medium when DNA is replicated rapidly (11, 12, 14). The *rpo∗* mutations suppress the loss of ppGpp (29, 30, 32, 34, 35) and all decrease the activity of rRNA promoters, thus potentially decreasing the rate of replication-transcription conflicts. Other stringent RNAP mutations, which do not have the *rpo∗* properties, may possibly lead to different changes on the transcriptomic level thus explaining their smaller effects on transcription-replication conflicts (29, 30, 32). Therefore, analysis of the effects of various classes of suppressor mutations on gene expression and RNAP distribution *in vivo*, in particular, on the transcription levels of the most highly transcribed rRNA operons, will be essential for understanding the exact nature of their diverse effects observed in various experimental systems.

## Experimental procedures

### Proteins

Substitutions K215E, Δ312-314, K789Q, and R1148H in the *rpoC* gene were obtained in pET29 containing the WT *E. coli rpoC* gene by site-directed mutagenesis and then transferred to the pVS10 expression vector encoding all RNAP subunits with a His6-tag in the C terminus of the β′ subunit. Substitutions Q148P, H447P, T563P, H1244Q, and G1260D in the *rpoB* gene were obtained in the pIA545 vector containing the WT *E. coli rpoB* gene by site-directed mutagenesis and then transferred into the vector pIA679 encoding all RNAP subunits with a His6-tag in the N terminus of the β subunit. WT *E. coli* core RNAP and its mutant variants were expressed in *E. coli* BL21(DE3) and purified as described previously (65, 78). The σ^70^ factor and DksA were expressed and purified from *E. coli* as described (37).

### Transcription *in vitro*

The schematics of all *in vitro* transcription experiments a shown in Fig. S1. For analysis of promoter complex stabilities, RNAP holoenzyme (50 nM core RNAP and 250 nM σ^70^ factor) was incubated with a PCR fragment (25 nM) containing the T7A1 promoter in transcription buffer (40 mM KCl, 10 mM MgCl_2_ and 40 mM Tris-HCl, pH 7.9 in most experiments) for 10 min at 37 °C. Heparin was added to 100 μg/ml, the samples were incubated for increasing time intervals, NTP substrates were added (100 μM ATP, GTP, CTP, 10 μM UTP with the addition of α-^32^P-UTP), and the reaction was stopped after 5 min at 37 °C by addition of an equal volume of stop-solution (8M urea, 20 mM EDTA, 2^x^TBE). Analysis of RNAP activity on the *rrnB* P1 promoter was performed using a pTZ19-derived plasmid containing the *rrnB* P1 promoter placed 88 nt upstream of the hisT terminator; the length of the control RNA I transcript encoded by the *ori* region in this plasmid is 108 to 110 nt (37). RNAP holoenzyme (200 nM core RNAP and 500 nM σ^70^ factor) was incubated with plasmid DNA (25 nM) in transcription buffer containing 150 mM KCl for 10 min at 37 °C. NTP substrates were added (200 μM ATP, GTP, CTP, 10 μM UTP with the addition of α-^32^P-UTP), and the reaction was performed for 10 min at 37 °C.

To measure the rate of transcription elongation, promoter complexes were formed on linear DNA containing the λP_R_ promoter and a 500 bp fragment of the *rpoB* gene (65, 79). Transcription was initiated with an incomplete NTP set (10 μM ApU, 25 μM ATP, GTP, 10 μM UTP with the addition of α-^32^P-UTP) for 7 min at 37 °C to obtain TECs stalled at the +26 template position. The samples were transferred to 20 °C, and all four NTPs were added (200 μM each, with 15 μg/ml heparin to prevent reinitiation), and the reaction was performed for increasing time intervals. DksA (final concentration 1 μM) and ppGpp (250 μM; TriLink BioTechnologies) were added prior to NTP addition, when indicated.

For analysis of the TEC stability, transcription was initiated on a linear DNA fragment containing the T7A1 promoter using an incomplete NTP set (10 μM ApU, 25 μM ATP, GTP, 10 μM CTP with the addition of α-^32^P-CTP) for 5 min at 37 °C to obtain TECs stalled at the +20 template position. Ni-NTA-agarose equilibrated in the transcription buffer was added to the samples (20 μl per 100 μl of the reaction volume), and the samples were stirred for 5 min at 37 °C. 4 M KCl was added to the final concentration of 1 M, the samples were incubated for increasing time intervals at 37 °C, 20 μl aliquots were removed, washed with 1 ml of the buffer containing 1 M KCl, then with 1 ml of the buffer containing 40 mM KCl, resuspended in 20 μl of the same buffer and 20 μl of the stop solution.

For analysis of intrinsic transcription termination, transcription was performed on a linear DNA fragment containing the T7A1 promoter and λ tR2 terminator. Promoter complexes were obtained at 37 °C as described above, NTPs were added (100 μM ATP, GTP, CTP, 10 μM UTP with addition of α-[^32^P]-UTP), and the reaction was stopped after 10 min. To determine the efficiency of transcription termination, the ratio of terminated (101 nt) to the sum of terminated and run-off (150 nt) products was calculated. Analysis of hisP pausing after promoter-dependent transcription initiation was performed in a similar way, using a linear DNA template containing the WT hisP pause sequence fused to the λ P_R_ promoter. TECs stalled at +26 template position were obtained after transcription initiation with an incomplete set of nucleotides (10 μM ApU, 25 μM ATP, GTP, 10 μM UTP with the addition of α-^32^P-UTP), then all four NTPs were added (10 μM GTP, 100 μM ATP, CTP, UTP), and RNA synthesis was performed for increasing time intervals at 37 °C.

Analysis of the kinetics of transcriptional pausing, nucleotide misincorporation, intrinsic RNA cleavage, and translesion RNA synthesis was performed in reconstituted TECs of various structures (Fig. S2). Unmodified DNA and RNA oligonucleotides were ordered from DNA Synthesis (Moscow), modified DNA oligonucleotides were purchased from TriLink BioTechnologies. For analysis of consP and hisP pausing, TECs stalled two nucleotides upstream of the expected pause site were obtained by stepwise reconstitution from the template DNA oligonucleotide, 5′-^32^P-labeled RNA oligonucleotide, core RNAP, and nontemplate DNA oligonucleotide in the transcription buffer as described previously (43, 79). For analysis of hisP pausing, antisense RNA was added during TEC assembly. NTP substrates (10 μM GTP and CTP for consP; 2 μM GTP, 100 μM UTP, and CTP for hisP) were added, and the reactions were stopped after increasing time intervals by the addition of the stop-solution.

Analysis of nucleotide misincorporation and mismatch extension was performed in fully complementary (rA-dT) and mismatched (rA-dG) TECs, respectively (Fig. S2). The TECs were reconstituted from synthetic oligonucleotides and core RNAP in the absence of MgCl_2_ as described above, either noncognate ATP (1 mM) or cognate CTP (1 mM) were added together with MgCl_2_ (10 mM) at 20 °C to initiate RNA extension, and the reactions were stopped after increasing time intervals. For analysis of intrinsic RNA cleavage, MgCl_2_ (10 mM) was added to the rA-dG TEC at 37 °C in the absence of NTP substrates, and the kinetics of RNA cleavage was monitored over time.

For analysis of translesion transcription, TECs were reconstituted using DNA oligonucleotides containing modified nucleotides downstream of the RNA 3′-end (Fig. S2) as described previously (50, 51). Briefly, 5′-P^32^-labeled RNA was mixed with the template and nontemplate DNA oligonucleotides (0.5 μM, 1 μM, and 5 μM final concentrations) in the transcription buffer at 65 °C and slowly cooled down to 25 °C. The samples were diluted to 10 nM RNA concentration, core RNAP was added to 25 nM, the samples were incubated for 10 min at 25 °C, NTPs were added to 100 μM, and the reactions were stopped after increasing time intervals. When indicated, DksA (2 μM) and ppGpp (200 μM) were added 5 min prior to NTP addition.

In all cases, RNA products were analyzed by 10%, 15%, or 23% PAGE and quantified with a Typhoon 9500 scanner using ImageQuant Software (GE Healthcare). The half-life times of T7A1 promoter complexes were calculated by fitting the data to the single-exponential equation A = A_max_×e x p(-*k*_obs_ × t), where A is the RNAP activity measured at a given time point, A_max_ is the projected maximal activity at zero time point, *k*_obs_ is the observed rate constant for promoter complex dissociation, and t_1/2_=ln2/*k*_obs_. The activities of WT and mutant RNAPs on the *rrnB* P1 promoter relative to the RNA I promoter were obtained by dividing the amounts of corresponding RNA products synthesized by each RNAP. The efficiencies of pausing in the consP and hisP TECs were calculated as the ratio of the paused RNA to the sum of the paused and read-through RNAs. The rates of the pause decay and the pause half-life times were calculated by fitting the data to the single-exponential equation P = P_max_ × exp(-*k*_obs_ × t) + B, where P is the pausing efficiency, P_max_ is the projected pausing at zero time point, *k*_obs_ is the observed rate constant for the pause decay, and B is the remaining fraction of permanently paused TECs; t_1/2_=ln2/*k*_obs_. To calculate the rates of intrinsic RNA cleavage, the efficiencies of RNA cleavage were calculated for each time point by dividing the amounts of the 13 nt cleavage product by the sum of 13 nt and 15 nt RNAs, and the data were fitted to the single-exponential equation C = C_max_ × (1 – exp(–k_obs_ × t)) + B, where C is the fraction of cleaved RNA, C_max_ is the maximal cleavage, B is a background level of cleavage at zero time point, and *k*_obs_ is the observed rate of the reaction. Statistical significance of the observed differences in the reaction rates was analyzed using Student's unpaired *t* test.

## Data availability

All experimental data are available from the corresponding authors upon request.

## Supporting information

This article contains supporting information.

## Conflict of interest

The authors declare that they have no conflicts of interest with the contents of this article.

## Supporting information

Miropolskaya_SI_FINAL
