# Supplementary material for: Suppressor mutations in Escherichia coli RNA polymerase alter transcription initiation but do not affect translesion RNA synthesis in vitro
Source: J Biol Chem. 2022 Jun 3;298(7):102099. doi: 10.1016/j.jbc.2022.102099 (PMC9254596; doi:10.1016/j.jbc.2022.102099)
Supplement: Miropolskaya_SI_FINAL [file mmc1.pdf]

Suppressor mutations in *Escherichia coli* RNA polymerase alter transcription initiation but do not affect translesion RNA synthesis *in vitro*

Nataliya Miropolskaya, Ivan Petushkov, Daria Esyunina, Andrey Kulbachinskiy

SUPPORTING INFORMATION

Figure S1 (next page). Schematics of the experiments performed in this study. Analysis of the promoter complex stability, the average rate of elongation, TEC stability, intrinsic transcription termination, transcriptional pausing (hisP pausing after promoter-dependent initiation; hisP and consP pausing in reconstituted TECs), nucleotide misincorporation and mismatch extension, intrinsic RNA cleavage and translesion RNA synthesis are shown from top to bottom.

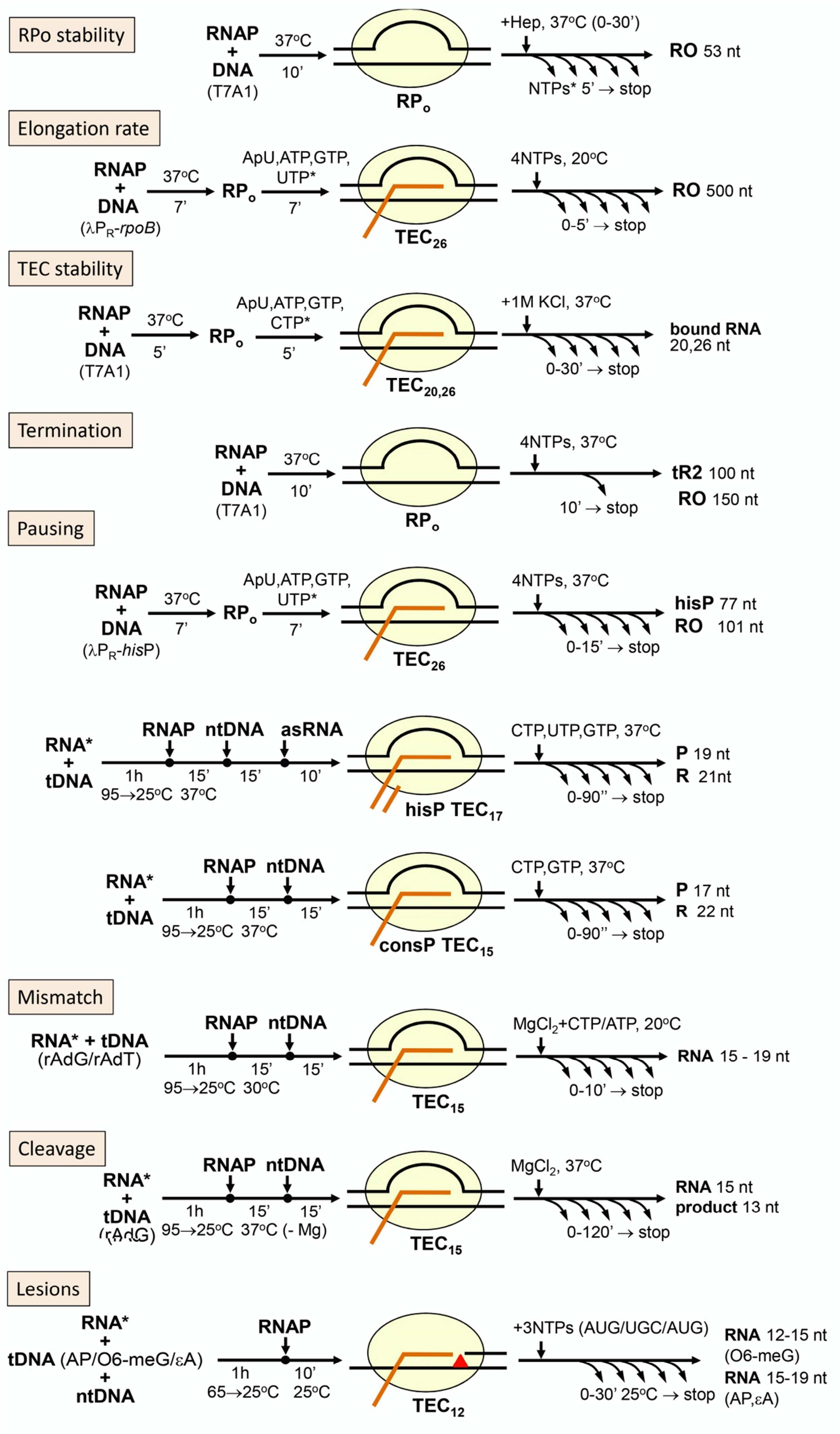
RPo stability
RNAP + DNA (T7A1)
37°C 10’
RPo
+Hep, 37°C (0-30’)
NTPs* 5’ → stop
RO 53 nt
Elongation rate
RNAP + DNA (λPR-rpoB)
37°C 7’
RPo
ApU,ATP,GTP, UTP* 7’
TEC26
4NTPs, 20°C
0-5’ → stop
RO 500 nt
TEC stability
RNAP + DNA (T7A1)
37°C 5’
RPo
ApU,ATP,GTP, CTP* 5’
TEC20,26
+1M KCl, 37°C
0-30’ → stop
bound RNA 20,26 nt
Termination
RNAP + DNA (T7A1)
37°C 10’
RPo
4NTPs, 37°C
10’ → stop
tR2 100 nt
RO 150 nt
Pausing
RNAP + DNA (λPR-hisP)
37°C 7’
RPo
ApU,ATP,GTP, UTP* 7’
TEC26
4NTPs, 37°C
0-15’ → stop
hisP 77 nt
RO 101 nt
RNA* + tDNA
RNAP ntDNA asRNA
1h 15’ 15’ 10’
95→25°C 37°C
hisP TEC17
CTP,UTP,GTP, 37°C
0-90’’ → stop
P 19 nt
R 21nt
RNA* + tDNA
RNAP ntDNA
1h 15’ 15’
95→25°C 37°C
consP TEC15
CTP,GTP, 37°C
0-90’’ → stop
P 17 nt
R 22 nt
Mismatch
RNA* + tDNA (rAdG/rAdT)
RNAP ntDNA
1h 15’ 15’
95→25°C 30°C
TEC15
MgCl2+CTP/ATP, 20°C
0-10’ → stop
RNA 15 - 19 nt
Cleavage
RNA* + tDNA (rAdG)
RNAP ntDNA
1h 15’ 15’
95→25°C 37°C (- Mg)
TEC15
MgCl2, 37°C
0-120’ → stop
RNA 15 nt
product 13 nt
Lesions
RNA* + tDNA (AP/O6-meG/εA) + ntDNA
RNAP
1h 10’
65→25°C 25°C
TEC12
+3NTPs (AUG/UGC/AUG)
0-30’ 25°C → stop
RNA 12-15 nt (O6-meG)
RNA 15-19 nt (AP,εA)

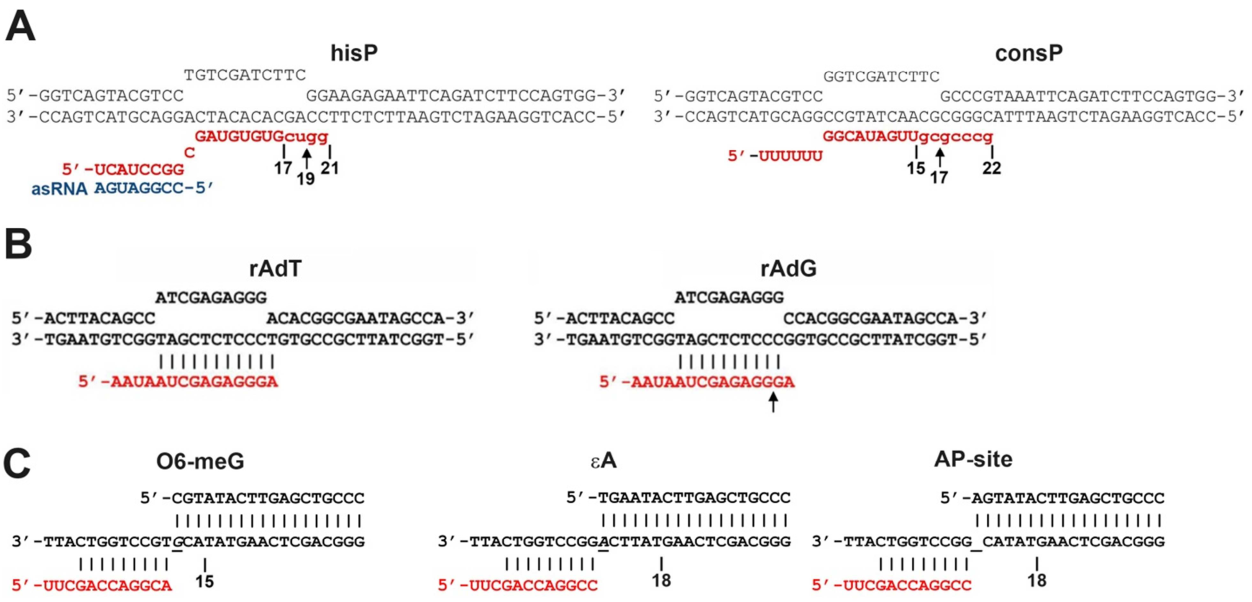

Figure S2. Schematics of the TECs reconstituted from synthetic oligonucleotides. (A) HisP and ConsP paused complexes. Positions of starting, paused and read-through transcripts are indicated. The antisense RNA oligonucleotide (asRNA) used for hisP reconstitution is shown in blue. (B) Correctly matched (rAdT) and mismatched (rAdG) complexes used for analysis of nucleotide misincorporation, mismatch extension and intrinsic RNA cleavage. (C) Reconstituted complexes containing DNA lesions (O6-meG, εA or AP-site) at indicated positions (underlined). Control TECs were obtained with corresponding unmodified nucleotides (G, A and T, respectively).

Table S1. Results of individual measurements of the pause half-life times and the rates of intrinsic RNA cleavage for wild-type and mutant RNAP variants.

RNAP	Reaction		
	**consP** ($t_{1/2}$, s)	**hisP** ($t_{1/2}$, s)	**cleavage** (k_{obs}, s^{-1})
WT	18.8, 28.9, 18.6	35.9, 52.8, 42.6	0.0083, 0.0029, 0.0049
Q148P	34.5, 35.5	70.6	0.0086, 0.0031, 0.0046
H447P	53.0, 44.5	73.9	0.0052, 0.0025, 0.0039
T563P	79.3, 81.6, 75.4	45.9, 44.9	0.0097, 0.0031, 0.0036
K215E	30.2, 28.6	46.5	0.0023, 0.0028, 0.0056
Δ312-314	36.1, 30.6	53.1	0.0024, 0.0025, 0.0056
K789Q	34.0, 29.0	36.4	0.0034, 0.0031, 0.0049
R1148H	38.2, 34.4	50.8	0.0018, 0.0032, 0.0034
H1244Q	46.2, 45.7, 40.5	114, 137, 110	0.0018, 0.0011, 0.0018
G1290D	34.4, 38.3	71.9, 65.3, 77.4	0.0026, 0.0011, 0.0011

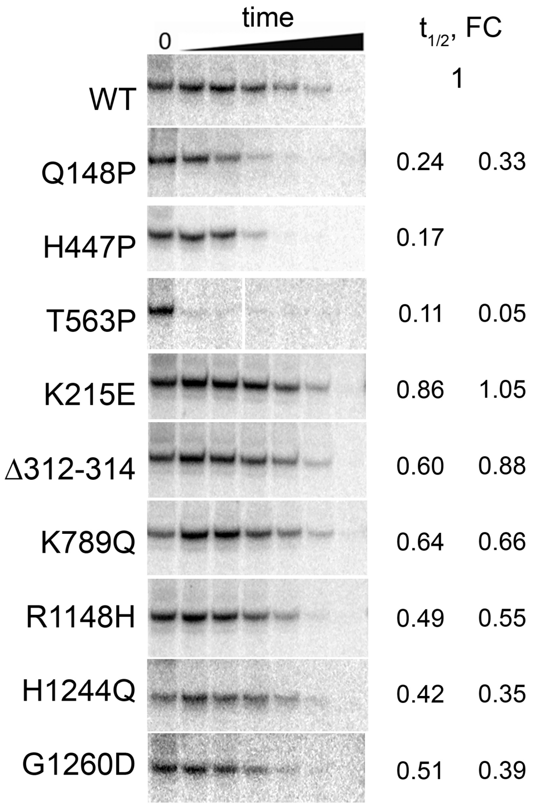

Figure S3. Analysis of stability of T7A1 promoter complexes of wild-type and mutant RNAPs. The second replicate of the experiment from Fig. 2A is shown. The H447P mutant was analyzed only once, the gel is taken from Fig. 2A. The samples for the T563P mutant were analyzed on two separate gels combined in one panel. RNAP activity was measured at increasing time intervals (0.5, 1, 2, 4, 10, 30 min) after heparin addition by analyzing run-off (RO) RNA synthesis. Fold-changes (FC) in the half-life times ($t_{1/2}$) of promoter complexes for the mutant RNAPs relative to the wild-type RNAP in the two experiments are shown on the right.

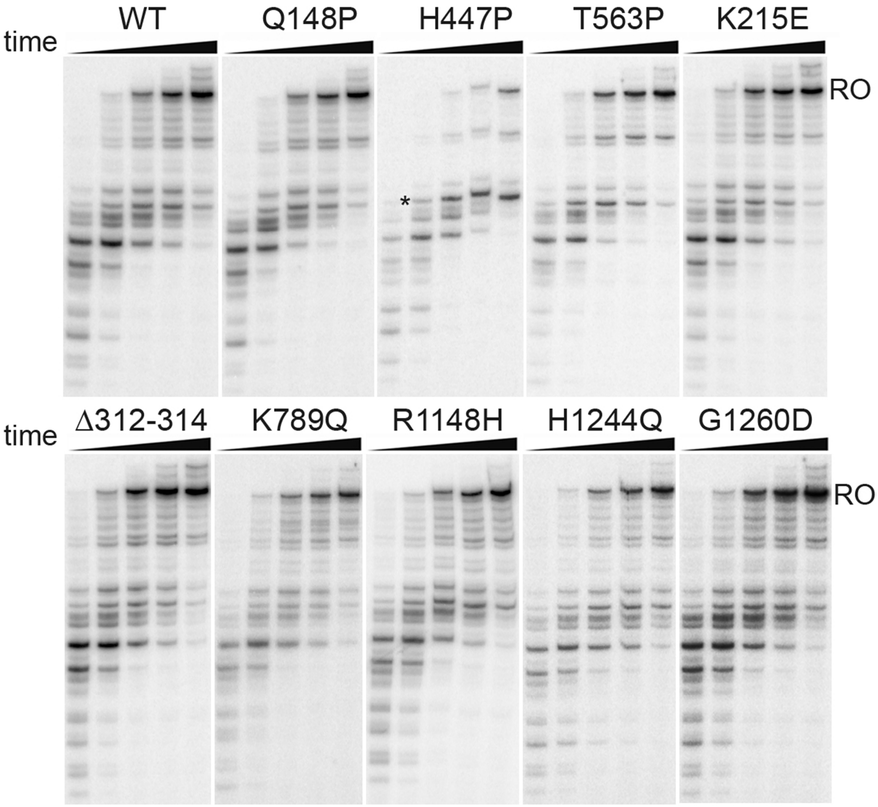

Figure S4. Transcription elongation by the mutant RNAPs in the presence of DksA and ppGpp. DksA and ppGpp were added to 1 μM and 250 μM, respectively. The kinetics of run-off (RO) RNA synthesis (0.5, 1, 2, 3, 5 min) was measured at 20 °C on the DNA template containing a 500 bp transcribed region downstream of the λP_R promoter. Transcriptional pause stimulated by the H447P substitution is indicated with an asterisk. The experiment was performed one time for each mutant (see Fig. 3 for a similar experiment performed without DksA/ppGpp).

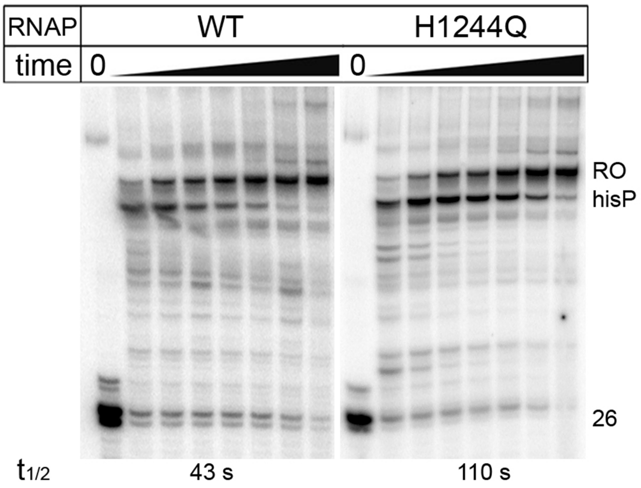

Figure S5. Analysis of hisP pausing by wild-type and H1244Q RNAPs. The reactions were performed on a DNA template containing the wild-type hisP sequence downstream of the λP_R promoter (10'', 20'', 40'', 1', 2', 5', 15' at 37 °C). Positions of the paused and full-length RNA transcripts are indicated. The pause half-life times are indicated below the gel (the results of a single measurement).

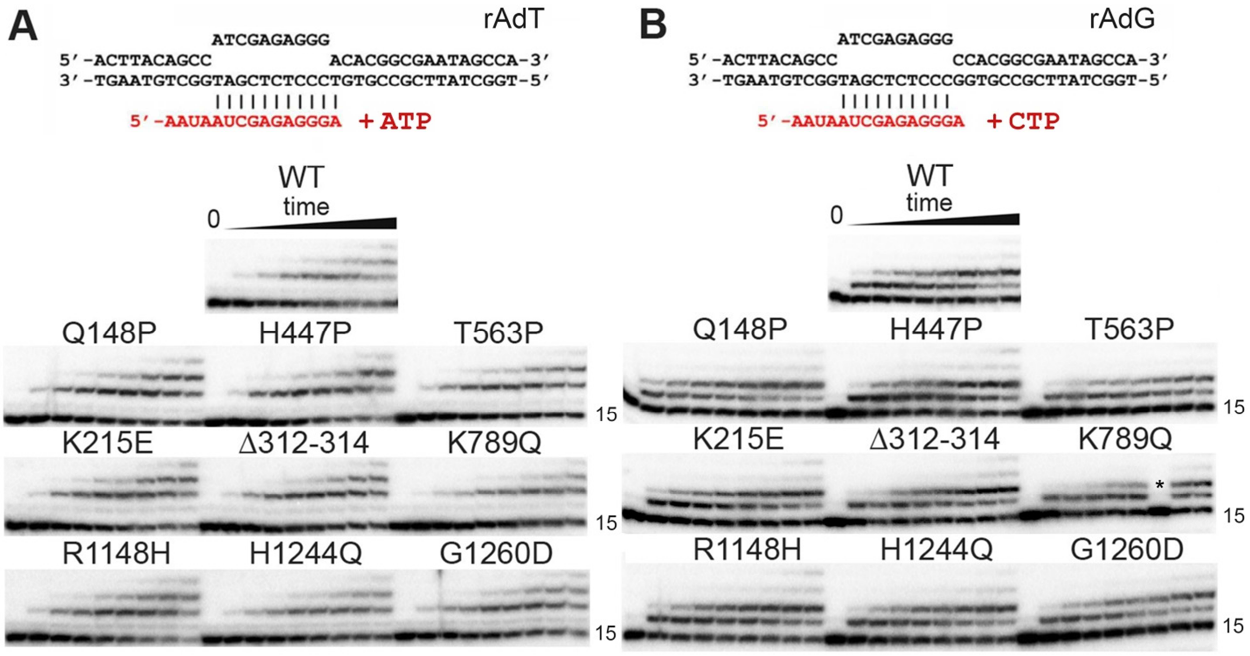

Figure S6. Analysis of nucleotide misincorporation (A) and mismatched RNA extension (B) by wild-type and mutant RNAPs. The reactions were performed in the correctly matched (rAdT) or mismatched (rAdG) reconstituted TECs, respectively, for 3", 10", 20", 40", 1', 2', 4' and 10' (the results of a single measurement for each RNAP are shown). The 2' time point for the K789Q mutant in panel (B) (indicated with an asterisk) accidentally was not measured and the lane contains starting RNA material. Positions of the starting RNA (15 nt) are indicated.

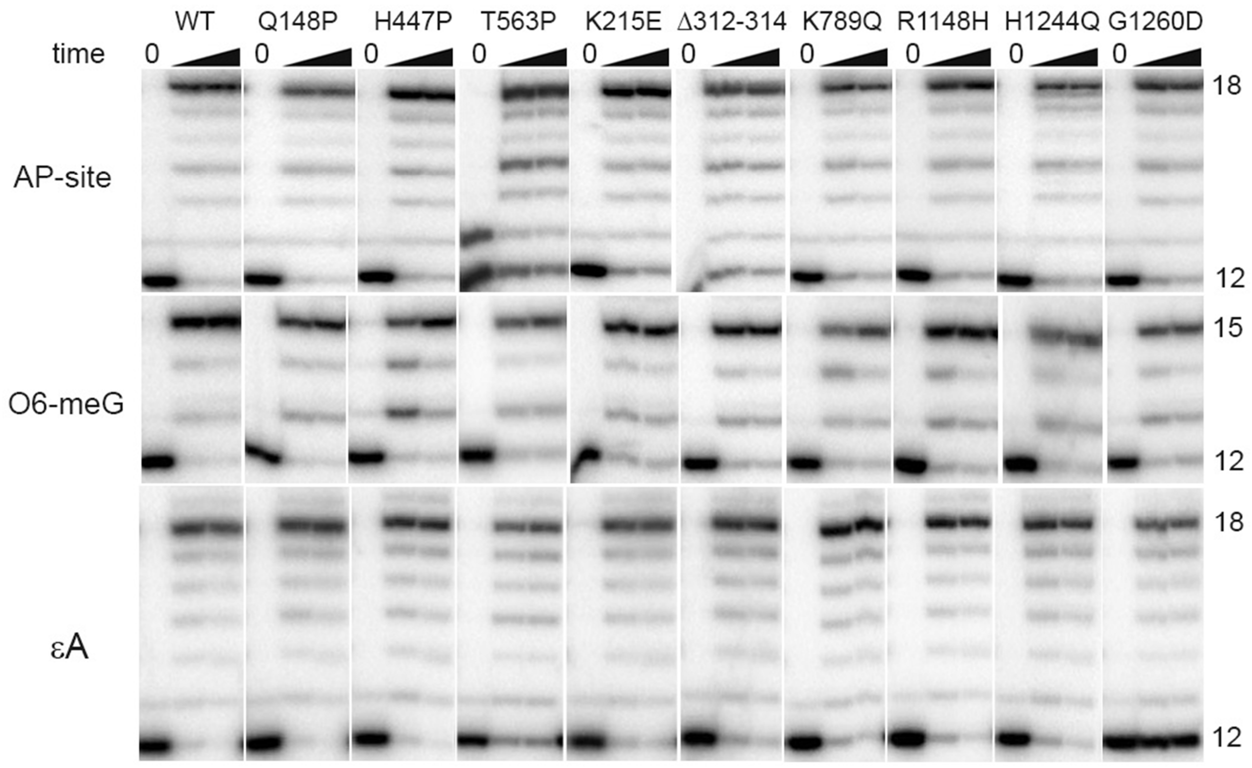

Figure S7. Kinetics of RNA synthesis on control DNA templates corresponding to each damaged template by the wild-type and mutant RNAPs. RNA extension was performed for 30” and 10’ in control TECs corresponding to the AP, O6-meG and εA templates (Fig. S2). For each TEC, an incomplete set of NTPs was added, resulting in transcription stalling at indicated positions (15 or 18 nt).

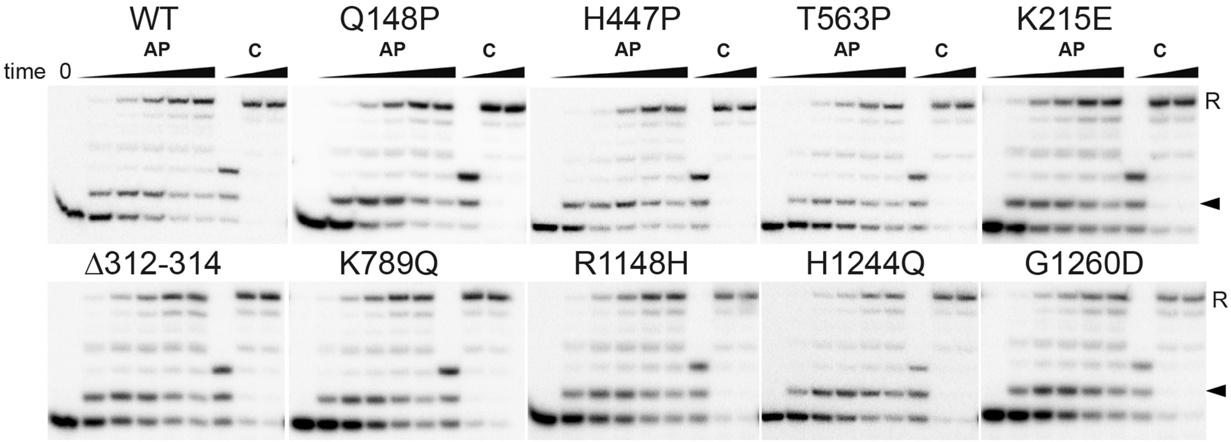

Figure S8. Kinetics of RNA synthesis on the AP-site template by the wild-type and mutant RNAPs in the presence of DksA and ppGpp. The reactions were performed for 10”, 30”, 1’, 3’ and 6’ on the AP-site template (AP) and for 10” and 3’ on the control (C) template (the results of a single measurement for each RNAP are shown). Positions of the AP site and the read-through RNA product (R) are indicated.
